# Bitterness quantification and simulated taste mechanism of theasinensin A from tea

**DOI:** 10.3389/fnut.2023.1138023

**Published:** 2023-05-09

**Authors:** Jian-yong Zhang, Hong-chun Cui, Zhi-hui Feng, Wei-wei Wang, Yun Zhao, Yu-liang Deng, He-yuan Jiang, Jun-feng Yin, Ulrich H. Engelhardt

**Affiliations:** ^1^Tea Research Institute, Chinese Academy of Agricultural Sciences, Hangzhou, China; ^2^Tea Research Institute of Hangzhou Academy of Agricultural Science, Hangzhou, China; ^3^Institute of Food Chemistry, Brunswick University of Technology, Braunschweig, Germany

**Keywords:** tea, theasinensin A, bitterness, threshold, molecular docking

## Abstract

Theasinensin A is an important quality chemical component in tea, but its taste characteristics and the related mechanism are still unclear. The bitterness quantification and simulated taste mechanism of theasinensin A were researched. The results showed that theasinensin A was significantly correlated with the bitterness of tea. The bitterness threshold of theasinensin A was identified as 65 μmol/L for the first time. The dose-over-threshold (DOT) value of theasinensin A was significantly higher than that of caffeine in black tea soup. The concentration-bitterness curve and time-intensity curve of theasinensin A were constructed. The bitterness contribution of theasinensin A in black tea was higher than in oolong and green tea. Theasinensin A had the highest affinity with bitterness receptor protein TAS2R16, which was compared to TAS2R13 and TAS2R14. Theasinensin A was mainly bound to a half-open cavity at the N-terminal of TAS2R13, TAS2R14, and TAS2R16. The different binding capacity, hydrogen bond, and hydrophobic accumulation effect of theasinensin A and bitterness receptor proteins might be the reason why theasinensin A presented different bitterness senses in human oral cavity.

## 1. Introduction

Tea is one of the three popular non-alcoholic beverages in the world. The consumption of tea is influenced by the taste of tea soup. The overall taste of tea infusion mainly comprises bitterness, astringency, umami, and sweetness (1). The bitterness is generally accepted as a sensation felt on the tongue caused by the interaction by the interaction between polyphenols, caffeine, and bitter receptors (2, 3), which greatly influenced the taste quality of tea infusion. The bitterness was an important quality attribute of tea infusion (4, 5). Objective evaluation of the bitterness of green tea soup has been reported (6–8). However, this research was developed based on a special taste compound evaluation system, which was difficult to be applied in other cases. Quantitatively analyzing the bitterness of different tea compound soups requires further investigation.

According to the metabonomic analysis method (9), it was speculated that theasinensins were strongly positively correlated with bitterness and astringency of green tea, which were not been evaluated by sensory evaluation. Theasinensins are a special kind of tea polyphenols, which could be detected in green tea (10), black tea (11, 12), white tea (13), oolong tea (14, 15), yellow tea (16), and pu'er tea (17). They comprise theasinensin A, theasinensin B, theasinensin C, theasinensin D, theasinensin E, theasinensin F, theasinensin G, and theasinensin H (18–20). Theasinensin A is the most abundant and important compound (21, 22). Due to the difficulty of large-scale preparation of theasinensin monomer, the taste characteristics and simulated taste mechanism of theasinensin A were still not clear. We successfully prepared theasinensin A monomer in batch (23), which laid a solid foundation for the taste evaluation and simulated taste mechanism of theasinensin A. Referring to the taste evaluation methods of tea (24–26), theasinensin A monomer was evaluated by sensory evaluation and had bitterness taste in this study. However, the threshold value, dose-over-threshold (DOT), concentration intensity relationship, and time–intensity relationship of theasinensin A were not clear.

Different flavor substances had different effects on the taste of tea soup. Ca^2+^ reduced the bitterness of green tea soup (6). Epicatechin gallate (ECG) and caffeine enhanced the bitterness of green tea soup (7). Different concentrations of theanine did not influence the bitterness of tea soup (27, 28). Benzene acetaldehyde and β-ionone enhance the sweetness of summer green tea (8). However, the influence of different concentrations of theasinensin A on the bitterness of different kinds of teas was still not clarified.

The mainstream view was that tea polyphenols had a bitter and astringent taste, which was mainly due to the interaction between tea polyphenols and salivary proteins (29–31). Different kinds of tea polyphenols showed different binding effects with receptor proteins. However, the spatial binding effect of theasinensin A molecule and receptor proteins was still unclear. Molecular docking was a very good tool to fastly simulate the binding mechanism between small molecules and receptor proteins, which was used to analyze the process of mutual recognition between two or more molecules through geometric matching and energy matching (32). The human body's taste receptor cells (TRCs), which were localized in the oral cavity in groups of cells called taste buds, sense bitterness (33). Each taste bud can comprise from 50 to 100 TRCs (34). These cells were characterized by the expression of members of the TASTE receptor type 2 (TAS2R) gene families, which encoded bitter taste receptors (35). In humans, there are 25 taste receptors (hTAS2Rs) (36), which were G protein-coupled receptors (GPCRs). Upon binding of bitter compounds to TAS2Rs, a bitter taste transduction response was triggered by the activation of Gα*βγ* protein subunits that subsequently activated several receptor potential channels. TAS2R13, TAS2R14, and TAS2R16 were three important human taste receptor proteins (36–38). It was necessary to analyze the interaction between theasinensin A and TAS2R13, TAS2R14, and TAS2R16 bitter receptor proteins by molecular docking, which was helpful to explore the bitterness mechanism of theasinensin A.

Based on the current research situation, we focus on the bitterness quantification character and simulated taste mechanism of theasinensin A. The threshold, DOT value, concentration intensity, and time intensity of theasinensin A monomer were studied. The DOT of theasinensin A, catechin, caffeine, and their contribution to the bitterness of tea soup were compared. Effects of different concentrations of theasinensin A monomer on the bitterness of green tea, black tea, and oolong tea were studied. The molecular docking method was employed to analyze the binding force and binding sites between theasinensin A and human TAS2R13, TAS2R14, and TAS2R16 bitter taste receptor proteins, so as to clarify the flavor quality characteristics and simulated taste mechanism of theasinensin A. The results might be useful for the establishment of quality evaluation methods for tea products and for taste improvement of tea beverages by accurate adjustment of the concentrations of theasinensin A and other taste substances.

## 2. Materials and methods

### 2.1. Materials

Epigallocatechin gallate (EGCG, ≥98%), gallocatechin gallate (GCG, ≥98%), epicatechin (EC, ≥98%), catechin (C≥98%), catechin gallate (CG, ≥98%), epicatechin gallate (ECG, ≥98%), and caffeine (≥98%) were purchased from Sigma, USA. Theasinensin A (≥98%) monomer was prepared at the Tea Research Institute of the Chinese Academy of Agricultural Sciences. Acetonitrile (chromatographically pure) and methanol (chromatographically pure) were purchased from Merck, Germany. Analytical reagents such as copper chloride, ascorbic acid, methanol, and phosphoric acid were purchased from Zhejiang Nade Scientific Instrument Co., Ltd., China. Black tea, oolong tea, and green tea samples were purchased from different areas in China. The Milli-Q ultrapure water was used.

### 2.2. Evaluation of bitterness threshold value and DOT of theasinensin A

The 200 μmol/mL mother liquor of theasinensin A monomer was prepared. Then the mother liquor was separately diluted to 40, 45, 55, 60, 65, 70, 75, 80, 85, 90, 95, 100, 105, 110, 115, 120, 125, 130, 135, 140, 145, and 150 μmol/L solution with pure water. The 3 mL diluted solution was then put into the evaluation cup. The sensory evaluators evaluated the taste characteristics of theasinensin A diluted solution. The minimum concentration of bitterness that could be felt by the evaluators was the bitterness threshold of theasinensin A. The ratio between the concentration of theasinensin A in tea soups and its bitterness threshold was the DOT of theasinensin A.

The theasinensin A solutions were scored at a room temperature of 25°C by 10 panelists composed of five men and five women between ages 20 and 50 years old, who were from the Tea Research Institute of the Chinese Academy of Agricultural Sciences. Ten panelists were trained to recognize and quantify bitterness using the caffeine soup with a concentration of 420 mg/L (7). Ten panelists were familiar with sensory experiments, and all of them achieved a certificate for tea-quality evaluation from the Tea Scientific Society of China. The bitterness was respectively divided into five regions, i.e., 0–2, 2–4, 4–6, 6–8, and 8–10. The 45 mL solution was put into a transparent glass cup at room temperature. Each panelist tasted 3 mL solution in their mouth and swirled it for 8–10 s before recording the bitterness score and subsequently spitting the solution. After 3–5 s, the bitterness score was recorded. The oral cavity was washed with pure water three times before the next sample was tasted. The interval between the two samples is 4 min. The evaluation process is divided into 20 sections. This method was also used in the sensory quality evaluation of the solution in the later chapters. Each panelist was given a nasal clip to prevent potential taste–odor interaction. The panelists were required to clean their palates with pure water before tasting the subsequent sample for preventing excessive sensory evaluation fatigue and delaying the effect of bitterness. The method of tasting and evaluating theasinensin A solution from low concentration to high concentration was adopted to avoid the delaying effect of bitterness. Panelists were also required to take a 45 min break every four samples to prevent sensory evaluation fatigue and adaptation.

### 2.3. Establishment of bitterness concentration intensity function model of theasinensin A

The 85, 110, 140, 155, 175, 350, 525, 700, 875, 1050, 1200, 1400, 1500, 1700, 1900, 2000, and 2250 μmol/L of theasinensin A were prepared. Sensory evaluators took the 3 mL sample solution each time and scored different concentrations of theasinensin A solution in turn. The reference substance was EGCG and caffeine. After that, the evaluator's mouth was cleaned with deionized water, they chewed soda biscuits, and waited for at least 30 s before tasting the next sample. The evaluation of each sample was completed within 5 min, and the maximum intensity was bitterness. Based on the sensory score and the concentration data of theasinensin A, the bitterness model of theasinensin A was established.

### 2.4. Establishment of time–intensity curves of the bitterness of theasinensin A

Theasinensin A solutions with concentrations of 200, 400, 600, 800, and 1000 μmol/L were prepared at 25°C. The sensory evaluators took 3 mL of the aforementioned solution, turned it in the mouth for 10 s, and then spit it out. At the same time, the bitterness intensity was recorded every 10 s, until the end of 240 s. After that, the mouth was cleaned two times with demonized water, chewed soda biscuits, and waited for at least 30 s before tasting the next sample. The time–intensity curve of the bitterness of theasinensin A was drawn.

### 2.5. Comparison of DOT of theasinensin A, catechins, and caffeine in tea soups

Nine tea samples (three black tea, three oolong tea, and three green tea) were chosen, including Dian black tea, Qimen black tea, Hainan black tea, Dahongpao oolong tea, Anxi oolong tea, Rougui oolong tea, Longjing green tea, Maojian green tea, and Biluochun green tea. Each tea sample (3.0 g) was brewed in boiling water (150 mL) for 5 min to obtain the soups used for chemical analyses. The brewed tea soups were naturally cooled at room temperature. Then tea soups were filtered through a 0.22 μm Millipore filter. The concentration of theasinensin A, catechins, and caffeine in tea soups was analyzed by HPLC (17). The ratio of the concentration of theasinensin A, catechins, and caffeine in tea soups and their bitterness threshold was the DOT of theasinensin A, catechins, and caffeine separately.

The tea soups were analyzed by HPLC (Shimadzu LC-20A; Shimadzu Corporation, Kyoto, Japan), which was equipped with a UV detector and a QDA mass spectrometry detector. The HPLC injection volume was 10 μL, and the column temperature was 30°C. The column was cosmosil 5C18-AR II (Nacalai Tesque Inc., Japan; 4.6 mm i.d. × 250 mm). Mobile phase A was 50 mmol/L phosphoric acid, and mobile phase B was 100% acetonitrile. The column temperature was maintained at 35°C. The gradient for solvent B was set as follows: 0–39 min, 4–30% B; 39–54 min, 30–75% B; 54–60 min, 75–4% B. The flow rate was 0.8 mL/min. The monitoring UV wavelength was set at 280 nm.

Mass spectrometry detection was conducted on a QDA in negative ion mode using full-scan recording in a mass range of m/z 50–950. A capillary voltage of 0.8 KV, sampling cone voltage of 15 V, source temperature of 120°C, and desolvation temperature of 600°C were used. The liquid chromatography combined with QDA mass spectrometry was used to determine the substrates and products, and quantitative analysis was performed using the internal standard method.

### 2.6. Effect of theasinensin A addition on the bitterness of tea soups

The 200, 1000, and 2000 μmol/L theasinensin A solutions were added to the aforementioned black tea, oolong tea, and green tea soups, respectively, as mentioned in Section 2.5. The theasinensin A solution was mixed with the tea soup solution at a volume ratio of 1:10. The sensory evaluators took 3 mL of the aforementioned solution, turned it in the mouth for 10 s, and then spat it out. The bitterness intensity score of the mixture infusion of theasinensin A and tea soups was recorded. The bitterness score of theasinensin A in tea soups was calculated by the bitterness regression equation mentioned in Section 3.2.

### 2.7. Molecular docking simulation of interactions between theasinensin A and human bitter receptor proteins

The small molecular structure of theasinensin A was prepared by the Autodock tool. The molecular format structure of theasinensin A was downloaded from ChemSpider software and then loaded into DS 3.5 client. Then, the structure of theasinensin A was optimized by energy. The atomic charge and atomic type were assigned by the Autodock tool 1.5.6 program. All rotatable keys of theasinensin A were set as flexible and were saved in the PDBQT format for molecular docking.

The structure of human taste receptor protein was prepared by the Autodock tool. The homologous models of TAS2R13, TAS2R14, and TAS2R16 human bitter taste receptors were constructed using C-C chemokine receptor type 5 (PDB ID 5UIW) as a template. A total of 100 independent structures of TAS2R13, TAS2R14, and TAS2R16 human bitter taste receptor protein was constructed by Modeler 9.18 soft. According to DOPE scoring, the conformation with the lowest numerical value was selected as the model conformation. Then, those were optimized by the AMBER force field, which was used as the final conformation for molecular docking. The protein file was loaded into the Autodock tool 1.5.6 program. The atomic charge and atomic type were assigned by the Autodock tool 1.5.6 program. All rotatable keys were set as flexible and were saved in PDBQT format for molecular docking.

Autodock vina1.1.2 was used for molecular docking of theasinensin A and human taste receptor protein. The ligand was set to be flexible. The receptor was set to be rigid. The docking box is located at the N-terminal of the protein. The scale of X- and Y-direction was 22 Å. The scale of the Z-direction was 24 Å. The conformation search precision was set to 32. The conformation with the highest score was selected as the molecular docking conformation for analysis.

### 2.8. Data processing

All results were recorded as mean ± standard deviation (three replicates). Significant differences between the means and stepwise regression were calculated by variance analysis with *post-hoc* analysis using SPSS version 25 (SPSS Inc., Chicago, IL, USA).

## 3. Results

### 3.1. Comparison of concentrations and DOT of taste substances in tea soups

The concentrations of taste substances in black tea, oolong tea, and green tea soups were analyzed (Table 1). The content of theasinensin A in black tea soup after 5 min extraction was significantly higher than that in oolong tea and green tea. In the black tea infusion, the leaching amount of theasinensin A was also significantly higher than that of epigallocatechin gallate (EGCG), gallocatechin gallate (GCG), epicatechin (EC), catechin (C), catechin gallate (CG), epicatechin gallate (ECG) and epigallocatechin (EGC), and gallocatechin (GC), and also significantly higher than that of theaflavin (TF), theaflavin monogallate (TFMG), and theaflavin 3,3′-digallate (TFDG), but lower than caffeine.

**Table 1:** Concentrations of taste substances in black tea, oolong tea, and green tea soups.

**Tastant**	**Black tea soup**			**Oolong tea soup**			**Green tea soup**		
	**Dian**	**Qimen**	**Hainan**	**Dahongpao**	**Anxi**	**Rougui**	**Longjing**	**Maojian**	**Biluochun**
TSA	187.30 ± 3.34 a	95.36 ± 0.31 b	64.24 ± 0.36 c	54.01 ± 1.43 d	5.45 ± 0.19 e	4.63 ± 0.15 f	ND	ND	ND
C	2.63 ± 0.11 f	1.70 ± 0.01 g	0.77 ± 0.00 h	7.35 ± 0.03 c	3.96 ± 0.03 e	5.01 ± 0.27 d	14.17 ± 0.05 b	16.12 ± 0.00 a	16.10 ± 0.09 a
EC	4.37 ± 0.27 g	5.45 ± 0.04 f	3.54 ± 0.16 h	1.70 ± 0.02 i	9.40 ± 0.03 d	7.30 ± 0.01 e	41.61 ± 0.19 c	82.04 ± 0.25 a	72.97 ± 0.30 b
EGCG	10.77 ± 0.21 f	8.91 ± 0.13 h	9.87 ± 0.18 g	43.16 ± 0.05 c	16.19 ± 0.04 e	21.56 ± 0.22 d	201.86 ± 0.15 a	129.22 ± 0.13 b	121.67 ± 0.29 b
GCG	1.15 ± 0.05 c	1.23 ± 0.01 c	0.68 ± 0.02 e	0.91 ± 0.01 d	0.08 ± 0.00 g	0.33 ± 0.01 f	64.85 ± 0.13 a	13.63 ± 0.08 b	12.73 ± 0.09 b
ECG	10.39 ± 0.03 d	2.67 ± 0.02 h	5.80 ± 0.05 e	10.70 ± 0.03 d	4.40 ± 0.00 f	3.72 ± 0.10 g	84.02 ± 0.15 c	93.01 ± 0.04 b	102.28 ± 0.56 a
CG	1.20 ± 0.02 c	2.23 ± 0.02 a	1.46 ± 0.01 b	1.39 ± 0.02 b	0.27 ± 0.00 g	0.59 ± 0.06 e	0.63 ± 0.01 e	0.70 ± 0.01 d	0.43 ± 0.01 f
Caffeine	197.08 ± 8.69 b	143.68 ± 0.45 e	119.47 ± 0.32 f	92.87 ± 0.51 g	25.09 ± 0.33 h	29.34 ± 0.16 i	238.31 ± 0.46 a	173.86 ± 0.26 d	182.28 ± 0.10 c

The unit of concentration is μmol/L. Different letters in the same column indicate significant differences between mean values (p < 0.05).

The bitterness threshold of theasinensin A was 65 μmol/L, which was identified for the first time in this study. Previous studies had shown that the theasinensins, flavonoid glycosides and organic acids, catechins, and proanthocyanidins were strongly positively correlated with bitterness and astringency by metabonomics analysis methods (6, 8). These results showed that the bitterness DOT value of theasinensin A was higher than caffeine in black tea (Table 2). However, the bitterness DOT value of theasinensin A was lower than caffeine in green tea. It was of great scientific significance to clarify the character and contribution of theasinensin A bitterness, which was useful to control tea beverage quality.

**Table 2:** Dose-over-threshold (DOT) of bitterness substances in black tea, oolong tea, and green tea soups.

**Tastant**	**Threshold (μmol/L)**	**Black tea soup**			**Oolong tea soup**			**Green tea soup**		
		**Dian**	**Qimen**	**Hainan**	**Dahongpao**	**Anxi**	**Rougui**	**Longjing**	**Maojian**	**Biluochun**
TSA	65	2.88 ± 0.09 a	1.47 ± 0.05 b	0.99 ± 0.05 c	0.83 ± 0.00 d	0.08 ± 0.00 e	0.07 ± 0.00 e	ND	ND	ND
C	860	ND	ND	ND	0.01 ± 0.00 c	ND	0.01 ± 0.00 c	0.02 ± 0.00 b	0.02 ± 0.00 b	0.03 ± 0.00 a
EC	930	ND	0.01 ± 0.00 d	ND	ND	0.01 ± 0.00 d	0.01 ± 0.00 d	0.08 ± 0.00 b	0.09 ± 0.00 a	0.04 ± 0.00 c
EGCG	190	0.06 ± 0.00 f	0.05 ± 0.00 g	0.05 ± 0.00 g	0.23 ± 0.01 c	0.09 ± 0.00 e	0.11 ± 0.00 d	0.64 ± 0.03 b	0.68 ± 0.00 b	1.06 ± 0.00 a
GCG	390	ND	ND	ND	ND	ND	ND	0.03 ± 0.00 b	0.03 ± 0.00 b	0.17 ± 0.00 a
ECG	180	0.06 ± 0.00 d	0.01 ± 0.00 g	0.03 ± 0.00 e	0.06 ± 0.00 d	0.02 ± 0.00 f	0.02 ± 0.00 f	0.57 ± 0.02 a	0.52 ± 0.00 b	0.47 ± 0.00 c
CG	170	0.01 ± 0.00 a	0.01 ± 0.00 a	0.01 ± 0.00 a	0.01 ± 0.00 a	ND	ND	ND	ND	ND
Caffeine	500	0.39 ± 0.01 b	0.29 ± 0.01 c	0.26 ± 0.01 c	0.19 ± 0.00 d	0.06 ± 0.00 e	0.06 ± 0.00 e	0.36 ± 0.01 b	0.35 ± 0.00 b	0.48 ± 0.00 a

Different letters in the same column indicate significant differences between mean values (p < 0.05). ND means that the substance is not detected. The bitterness threshold of C, EC, EGCG, GCG, ECG, CG, and caffeine was cited from Xu et al. (8).

### 3.2. Concentration–taste intensity curves and time–intensity curves of the bitterness of theasinensin A

**Figure 1:**
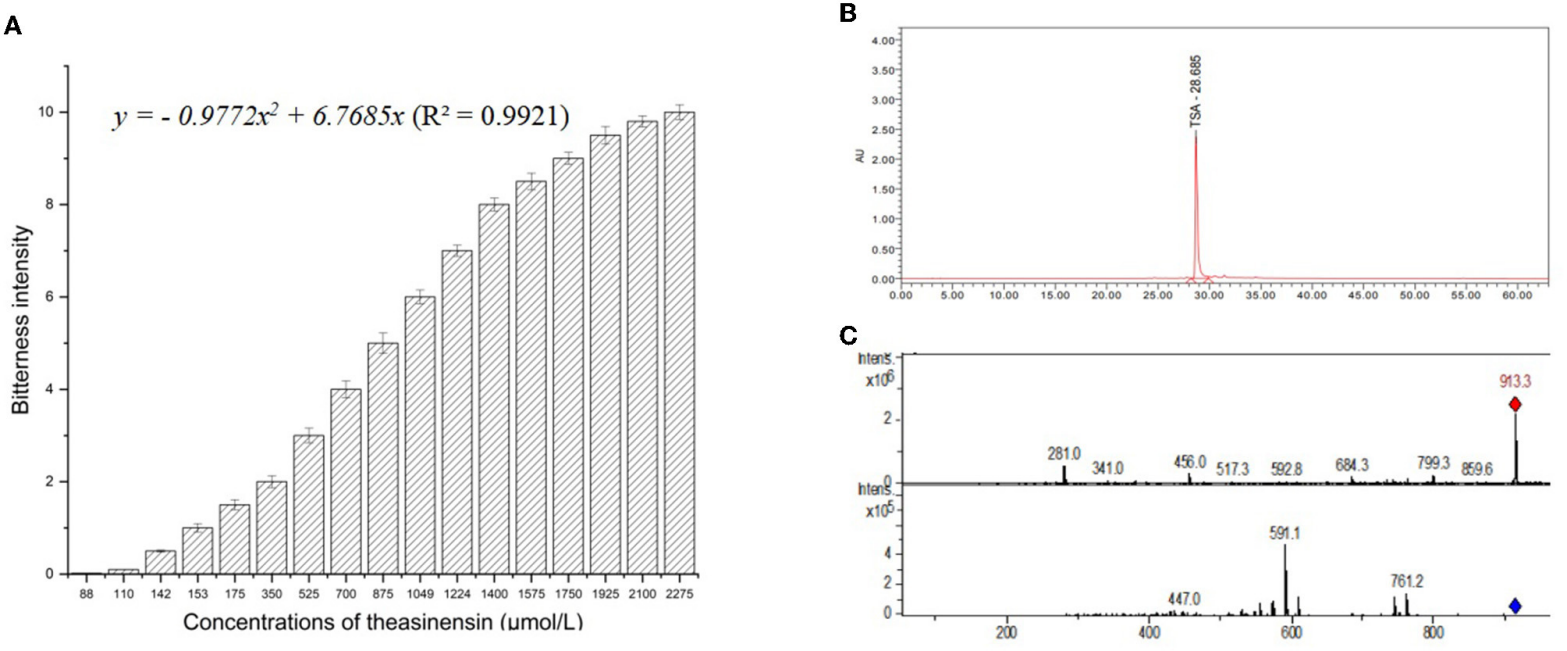
Bitterness intensity model **(A)**, high-performance liquid chromatogram **(B)**, mass spectrogram of theasinensin A **(C)**.

The concentration–taste intensity curves of bitterness (Figure 1A) and liquid chromatography–mass spectrometry of theasinensin A (Figure 1C) were established. The concentration–intensity curves for the bitterness of the theasinensin A fit the cubic functions well and have *R*^2^ values >0.99. The regression functions equation for the bitterness of theasinensin A was as follows:

Theasinensin A bitterness intensity regression function (Figure 1A): *y* = −*0.9772x*^2^ + *6.7685x* (R^2^ = 0.9921).

*y* was theasinensin A bitterness sensory score, and *x* was theasinensin A concentration (μmol/L).

**Figure 2:**
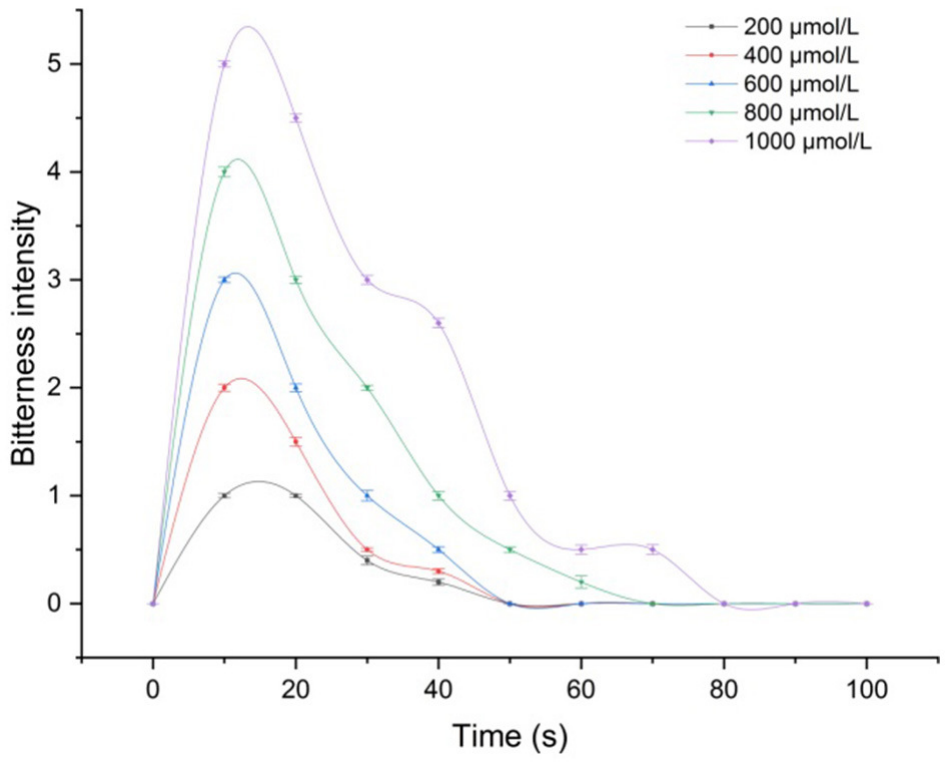
Time-intensity curve of the bitterness of theasinensin A.

The time-intensity curve of bitterness (Figure 2) of theasinensin A was drawn. In different concentrations of theasinensin A solution in the human oral cavity for 10 s, the bitterness feeling was the strongest, which gradually decreased (Figure 2). Low concentration of theasinensin A solution had a shorter bitterness relaxation time (5 s) in the human oral cavity. With the increase of concentration of theasinensin A solution, the bitterness relaxation time of theasinensin A solution in the human oral cavity was gradually prolonged, and the relaxation time decreased with the concentration gradient.

The bitterness of theasinensin A in the black tea, oolong tea, and green tea soups was calculated by the theasinensin A bitterness intensity regression functions (Table 3). The calculated bitterness score of theasinensin A in black tea soup was significantly higher than those in green tea soup and oolong tea soup. Moreover, the calculated bitterness score of theasinensin A in oolong tea soup was significantly higher than those in green tea soup. The bitterness contribution of theasinensin A in black tea, oolong tea, and green tea showed a similar trend to the calculated bitterness scores of theasinensin A. In this study, the contributions of theasinensin A to the bitterness of black tea soup was 24.04 ± 0.56 % – 83.64 ± 4.16%. The contributions of theasinensin A to the bitterness of oolong tea soup were 1.26 ± 0.05%−15.84 ± 0.87%.

**Table 3:** Bitterness score and contribution of theasinensin A(TSA) in tea soups.

**Item**	**Black tea soup**			**Oolong tea soup**			**Green tea soup**		
	**Qimen**	**Dian**	**Hainan**	**Anxi**	**Dahongpao**	**Rougui**	**Biluochun**	**Maojian**	**Longjing**
Bitterness score of TSA	0.64 ± 0.01 b	0.92 ± 0.04 a	0.43 ± 0.01 c	0.00 ± 0.00 e	0.36 ± 0.02 d	0.02 ± 0.00 f	0.00 ± 0.00 g	0.00 ± 0.00 g	0.00 ± 0.00 g
Bitterness score ratio of TSA to tea soup (%)	42.62 ± 1.32 b	83.64 ± 4.16 a	24.04 ± 0.56 c	1.85 ± 0.12 e	15.84 ± 0.87 d	1.26 ± 0.05 f	0.00 ± 0.00 g	0.00 ± 0.00 g	0.00 ± 0.00 g

Score of TSA in tea soups was calculated by the TSA bitterness regression equation of section 3.2. Different letters in the same row indicate significant differences between mean values (p < 0.05).

### 3.3. Evaluation of theasinensin A addition on the bitterness of tea soups

**Figure 3:**
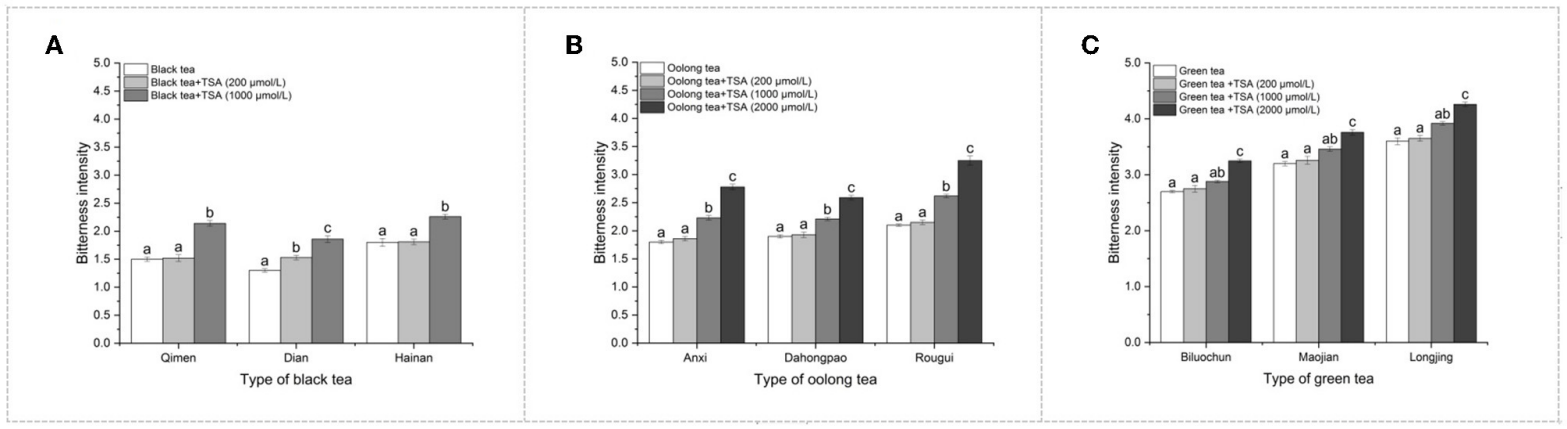
Effect of theasinensin A addition on the bitterness of tea soups. TSA was added to black tea **(A)**, oolong tea **(B)**, and green tea **(C)** to study the effect on the bitterness of tea soup. [Nore: 200 μmol/L, 1,000 μmol/L, and 2,000 μmol/L of TSA concentrations correspond to 1, 5, and 10 of bitterness sensory intensity score of TSA respectively. ^a, b, c^Different letters in the same column indicate significant differences between mean values (*p* < 0.05)].

In order to clarify the effect of theasinensin A on the bitterness of tea soups, different amounts of theasinensin A were added to the black tea, oolong tea, and green tea soups. As shown in Figure 3, in addition to Dianhong black tea soup, a low concentration of 200 μmol/L of theasinensin A with a bitterness of score 1.0 had no significant effect on the bitterness of black tea, oolong tea, and green tea soups. With the increase of theasinensin A concentration, the intensity of theasinensin A on the increase of bitterness of black tea, green tea, and oolong tea soups also increased (Figures 3A–C). The high concentration of 2,000 μmol/L of theasinensin A with a bitterness of score 10.0 had a high significant effect on the bitterness of black tea, oolong tea, and green tea soups (Figures 3A–C), while the high concentration of 1,000 μmol/L of theasinensin A with the bitterness of score 5.0 had a high significant effect on the bitterness of oolong tea and green tea soups.

Comparing the effect of theasinensin A addition on the bitterness of different types of tea, the enhancement effect of theasinensin A on the bitterness of oolong tea was stronger than that of black tea and green tea, especially at high concentrations. The results showed that the effect of theasinensin A on the bitterness of black tea, oolong tea, and green tea was significant, and a high concentration of theasinensin A affected bitterness of tea soup. In a word, it could be found that the scores and contribution of theasinensin A bitterness in different types of black tea, oolong tea, and green tea were different, which might be related to the complexity of the tea soup system. Due to space limitations, the interaction of various components of the tea soup needed to be further studied.

### 3.4. Binding capacity of theasinensin A to different human bitter taste receptor proteins

The TAS2R13, TAS2R14, and TAS2R16 bitter taste receptors were employed to bind theasinensin A for clarifying its binding ability and binding region. Theasinensin A had different binding abilities to the three human bitter receptor proteins. The binding capacity of theasinensin A to human taste receptor protein TAS2R13 was−10 kcal/mol. The binding capacity of theasinensin A to human taste receptor protein TAS2R14 was −8.6 kcal/mol. The binding capacity of theasinensin A to human taste receptor protein TAS2R16 was−10.9 kcal/mol. It could be seen that the binding ability of theasinensin A and TAS2R16 is relatively strong. TAS2R16 is a member of a family of candidate taste receptors that are members of the G protein-coupled receptor superfamily. These family members are specifically expressed by taste receptor cells of the tongue and palate epithelia. Each of these apparently intronless genes encodes a 7-transmembrane receptor protein, functioning as a bitter taste receptor. TAS2R13 and TAS2R14 also belong to the family of candidate taste receptors that are members of the G-protein-coupled receptor superfamily, which often exist in the skin cells, heart tissue, lung tissue, and airway smooth muscle cells. The cDNA gene expression band size of TAS2R16 was 419 bp, which was less than TAS2R13 (742 bp) and TAS2R14 (796 bp). The reason why theasinensin A had different binding abilities than the three human bitter receptors might be that theasinensin A more easily binds to smaller tongue and palate epithelia bitter receptor proteins.

**Figure 4:**
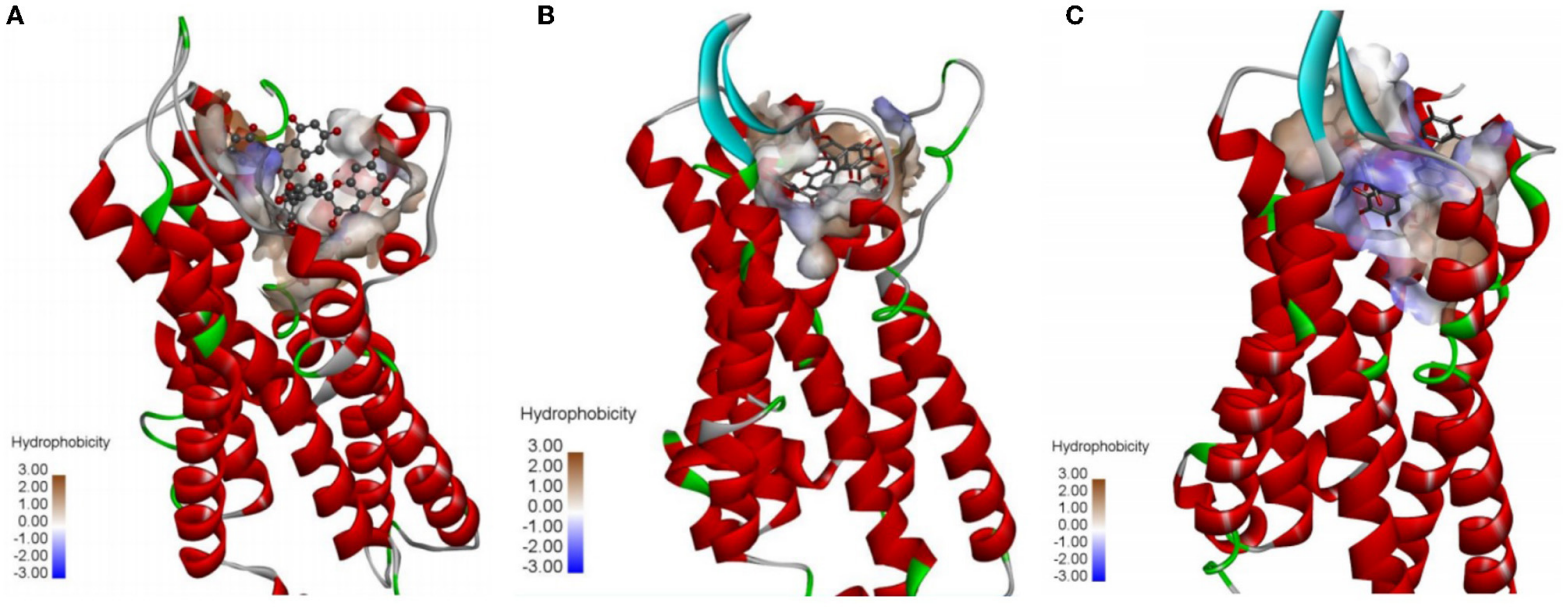
Overall binding of theasinensin A to human taste receptor protein TAS2R13 **(A)**, TAS2R14 **(B)**, and TAS2R16 **(C)**.

As shown in Figure 4, theasinensin A mainly bound to the N-terminal cavity of human taste receptor protein TAS2R13, TAS2R14, and TAS2R16 (Figures 4A–C). The N-terminal cavity had large hydrophobicity. The interaction between theasinensin A and the three bitter receptor proteins was mainly hydrophobic.

### 3.5. Binding action of theasinensin A to different human bitter taste receptor proteins

**Figure 5:**
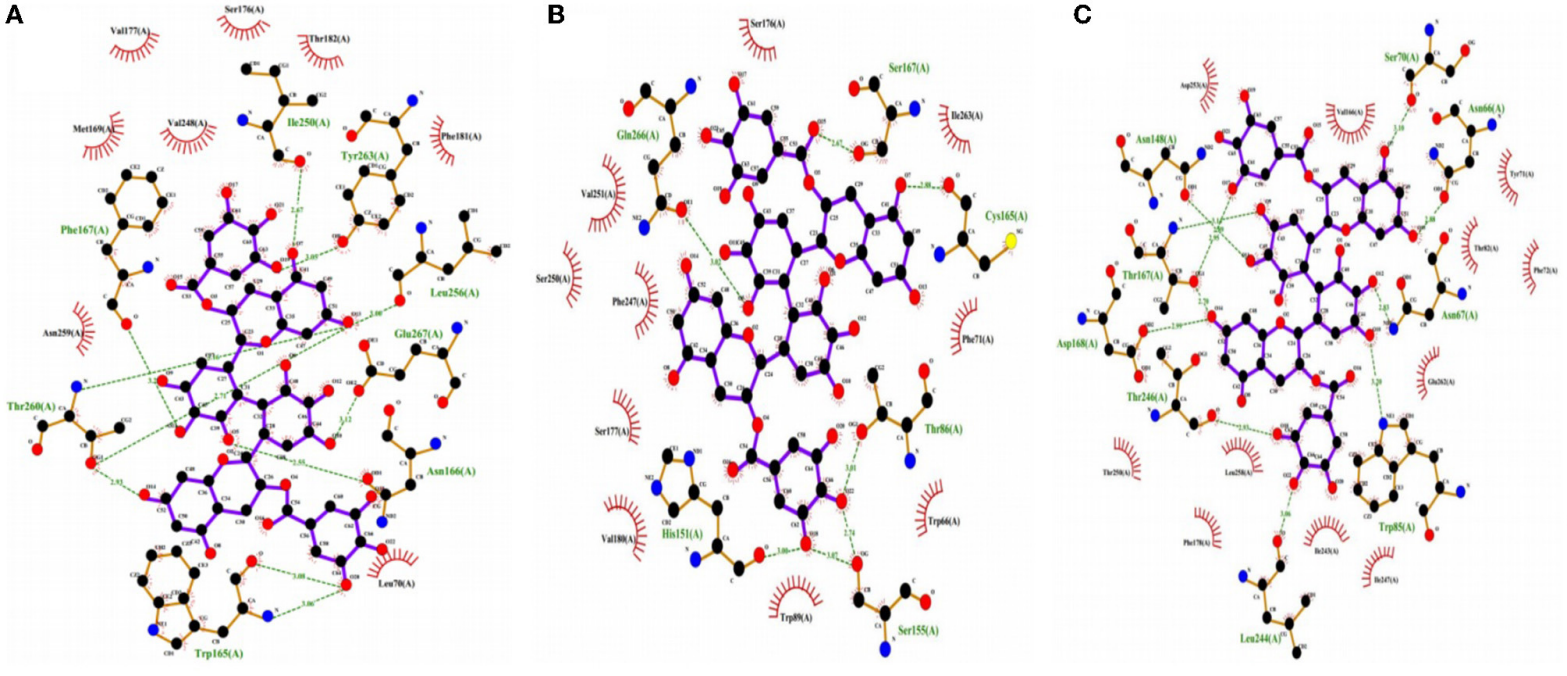
Binding sites of theasinensin A and human taste receptor protein TAS2R13 **(A)**, TAS2R14 **(B)**, and TAS2R16 **(C)**.

As shown in Figure 5, the binding action of theasinensin A to different human bitter taste receptor proteins was different. The molecular structure of theasinensin A had multiple hydroxyl groups, which could bind with TAS2R13, TAS2R14, and TAS2R16 bitter receptor proteins by 11, 7, and 11 hydrogen bonds, respectively. In addition, the molecular structure of theasinensin A had multiple hydrophobic benzene rings, which could have different hydrophobic accumulation with the binding N-terminal cavities of TAS2R13, TAS2R14, and TAS2R16 bitter receptor proteins. The difference in hydrogen bond binding and hydrophobic stacking between different bitter receptor proteins and theasinensin A was closely related to the difference in the recognition ability of theasinensin A by different bitter receptor proteins. The TAS2R16 bitter receptor protein binding N-terminal cavity that could hydrophobically accumulate with theasinensin A includes amino acids such as VAL166, TYR71, PHE72, LEU258, PHE178, ILE243, and ILE247. The TAS2R13 bitter receptor protein binding N-terminal cavity that could hydrophobically accumulate with theasinensin A includes amino acids such as VAL248, PHE181, ILE167, and TYR263. The TAS2R14 bitter receptor protein binding N-terminal cavity that could hydrophobically accumulate with theasinensin A includes amino acids such as ILE263, VAL251, PHE247, PHE71, VAL180, and TRP89. The number of amino acid residues in the TAS2R16 binding N-terminal cavity that could hydrophobically accumulate with theasinensin A was more than that in TAS2R13 and TAS2R14. The types of amino acid residues were also different. In a word, the aforementioned results suggested that a stronger interaction existed between theasinensin A and TAS2R16 bitter receptor protein.

According to the aforementioned molecular dynamics simulation results, it could be predicted that the theasinensin A would have hydrogen bonding and hydrophobic accumulation with bitter receptor proteins such as TAS2R16 in human oral cells, conjugates of which would be deposited on the human oral mucosa and tongue, thus made people perceive the bitter taste of theasinensin A. This might be the bitter taste presentation mechanism of TSA based on the binding of bitter receptor proteins.

## 4. Conclusion

Theasinensin A was a very important tea taste substance, which has different effects on different kinds of tea flavors. It was necessary to study the taste characteristics and simulated taste mechanisms. In this study, the bitterness threshold of theasinensin A was 65 μmol/L. The dose-over-threshold (DOT) value of theasinensin A was significantly higher than that of catechins and theaflavins in black tea soup. The model of the intensity of bitterness of theasinensin A was established, and the correlation coefficient was 0.9921. The bitterness time-intensity curve of theasinensin A was established. The taste contribution of theasinensin A in black tea was higher than in oolong tea and green tea. Theasinensin A had the highest affinity with taste receptor protein TAS2R16, which was−10.9. Theasinensin A was mainly bound to a half-open cavity at the N-terminal of TAS2R13, TAS2R14, and TAS2R16. There were multiple hydrophobic benzene rings of theasinensin A, which could form a hydrophobic effect and facilitate the binding of molecules. The bitterness characteristics analysis and effect of theasinensin A on the taste of different kinds of tea soup would help to enrich the theoretical basis of the bitterness chemistry of tea soup.

Bitterness is a basic flavor of tea, which was usually assessed by sensory analysis. Future studies should be focused on the change mechanism of bitterness taste characteristics of theasinensin A during human oral processing. In addition, the transmission regularity of theasinensin A bitter taste in tea and tea food processing was another key scientific problem. According to the formation mechanism of theasinensin A bitterness taste, it was also worth paying attention to the bitterness taste masking techniques such as the regulation of complex reaction of polyphony-protein, addition of flavoring agents, and blocking of trigeminal nerve conduction pathway.

## Data availability statement

The datasets presented in this study can be found in online repositories. The names of the repository/repositories and accession number(s) can be found in the article/Supplementary material.

## Author contributions

J-yZ: conceptualization and methodology. J-yZ, YZ, J-fY, Y-lD, and H-yJ: resources and funding acquisition. J-yZ, H-cC, and Z-hF: data curation. J-yZ and H-cC: writing—original draft preparation. J-yZ, W-wW, and UE: writing—review and editing. J-fY: supervision. All authors have read and agreed to the published version of the manuscript.
