# Supplementary material for: Bitterness quantification and simulated taste mechanism of theasinensin A from tea
Source: Front Nutr. 2023 May 9;10:1138023. doi: 10.3389/fnut.2023.1138023 (PMC10203438; doi:10.3389/fnut.2023.1138023)

Supplementary materials

The representative HPLC chromatographs of black tea:

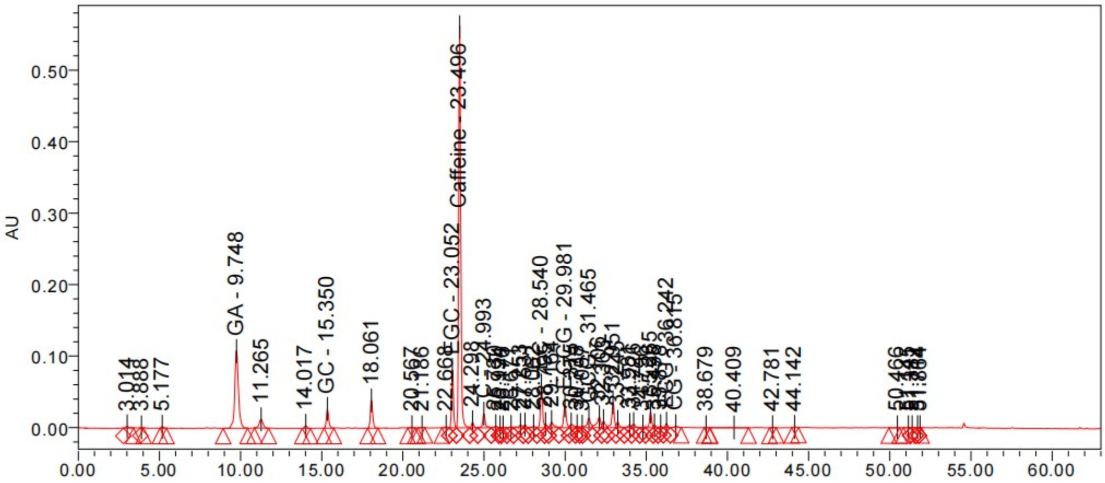

The representative HPLC chromatographs of oolong tea:

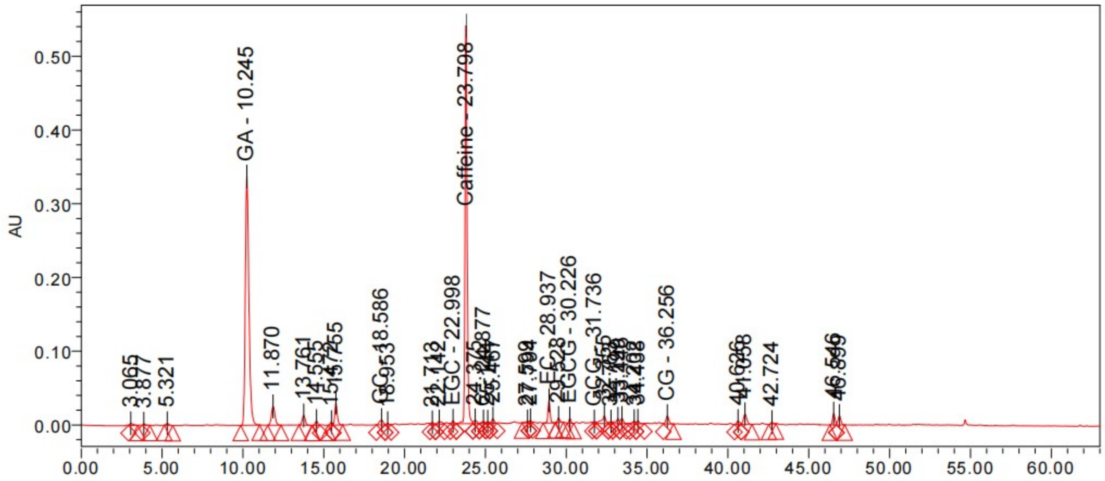

The representative HPLC chromatographs of green tea:

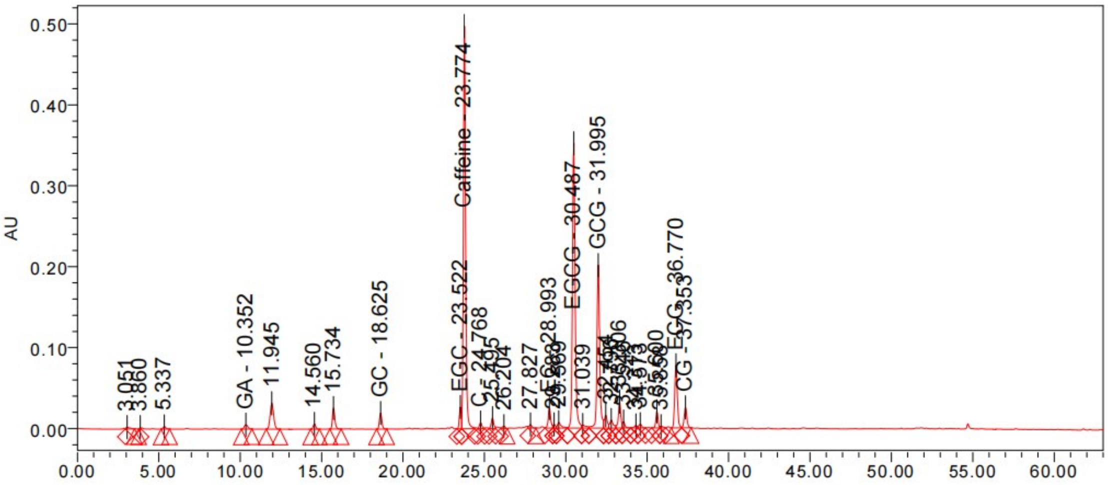

Supplement: Supplementary file 1 [file Data_Sheet_1.pdf]
